# Knockdown delta-5-desaturase in breast cancer cells that overexpress COX-2 results in inhibition of growth, migration and invasion via a dihomo-γ-linolenic acid peroxidation dependent mechanism

**DOI:** 10.1186/s12885-018-4250-8

**Published:** 2018-03-27

**Authors:** Yi Xu, Xiaoyu Yang, Tao Wang, Liu Yang, Yu-Ying He, Keith Miskimins, Steven Y. Qian

**Affiliations:** 1 Department of Pharmaceutical Sciences, North Dakota State University, Fargo, ND 58108 USA; 2 Department of Transplantation, Mayo Clinic Florida, Jacksonville, FL 32224 USA; 3 Department of Medicine, Section of Dermatology, University of Chicago, Chicago, USA; 4 Cancer Biology Research Center, Sanford Research, Sioux Falls, SD 57104 USA

**Keywords:** COX-catalyzed DGLA peroxidation, delta-5-desaturase, D5D knockdown MDA-MB 231 and 4 T1 cells, breast cancer growth, migration and invasion

## Abstract

**Background:**

Cyclooxygenase-2 (COX-2), the inducible COX form, is a bi-functional membrane-bound enzyme that typically metabolizes arachidonic acid (downstream ω-6 fatty acid) to form 2-series of prostaglandins known to be involved in cancer development. Overexpression of COX-2 has been found in a majority of breast carcinomas, and has also been associated with increased severity and the development of the metastasis. Our lab recently demonstrated that COX-2 can also metabolize dihomo-γ-linolenic acid (DGLA, a precursor of ω-6 arachidonic acid) to produce an anti-cancer byproduct, 8-hydroxyoctanoic acid (8-HOA) that can inhibit growth and migration of colon and pancreatic cancer cells. We thus tested whether our strategy of knocking down delta-5-desaturase (D5D, the key enzyme that converts DGLA to arachidonic acid) in breast cancer cells overexpressing COX-2 can also be used to promote 8-HOA formation, thereby suppressing cancer growth, migration, and invasion.

**Methods:**

SiRNA and shRNA transfection were used to knock down D5D expression in MDA-MB 231 and 4 T1 cells (human and mouse breast cancer cell lines expressing high COX-2, respectively). Colony formation assay, FITC Annexin V/PI double staining, wound healing and transwell assay were used to assess the effect of our strategy on inhibition of cancer growth, migration, and invasion. GC/MS was used to measure endogenous 8-HOA, and western blotting was performed to evaluate the altered key protein expressions upon the treatments.

**Results:**

We demonstrated that D5D knockdown licenses DGLA to inhibit growth of breast cancer cells via promoting formation of 8-HOA that can inhibit histone deacetylase and activate cell apoptotic proteins, such as procaspase 9 and PARP. Our strategy can also significantly inhibit cancer migration and invasion, associated with altered expression of MMP-2/− 9, E-cadherin, vimentin and snail. In addition, D5D knockdown and DGLA supplementation greatly enhanced the efficacy of 5-fluorouracil on breast cancer growth and migration.

**Conclusions:**

Consistent to our previous studies on colon and pancreatic cancer, here we demonstrate again that the high level of COX-2 in breast cancer cells can be capitalized on inhibiting cancer growth and migration. The outcome of this translational research could guide us to develop new anti-cancer strategy and/or to improve current chemotherapy for breast cancer treatment.

**Electronic supplementary material:**

The online version of this article (10.1186/s12885-018-4250-8) contains supplementary material, which is available to authorized users.

## Highlights

- High level of COX-2 could be exploited to inhibit breast cancer cell growth and migration
- 8-hydroxyoctanoic acid, generated from D5D knockdown and DGLA supplementation, serves HDAC inhibitor, causes DNA damage, and suppresses breast cancer growth, migration and invasion
- D5D knockdown warranties DGLA’s anti-cancer activity and improves anti-cancer effect of chemotherapy in breast cancer cells

## Background

Breast cancer is the leading cause of cancer death worldwide in women and about 90% deaths from breast cancer are a consequence of metastasis. A variety of therapeutic and nutritional approaches including chemotherapy, targeted therapy, and ω-3 fatty acid dietary manipulation have been studied for breast cancer treatment [1–12]. ω-6 s fatty acids which are inevitable and more pervasive in our daily diet (ratio of ω-6 s vs. ω-3 s in traditional western diet is between ~ 10:1 and 30:1) have received much less research attention in cancer therapy, mainly due to deleterious metabolites (2-series prostaglandins, e.g., PGE2) formed from cyclooxygenase (COX)-catalyzed peroxidation of arachidonic acid (AA, a downstream ɷ-6 fatty acid) [13–19]. In contrast, COX-2 substrates, including eicosapentaenoic acid and docosahexaenoic acid (two ω-3 fatty acids) and dihomo-γ-linolenic acid (perhaps an exceptional ω-6 fatty acid), have shown some inhibitory effects on cancer cell growth and metastasis, most likely due to their competition with AA for the COX-2 peroxidation to limit PGE2 formation [6–12, 20–23].

COX is a bi-functional membrane bound and lipid peroxidizing enzyme with two isoforms, a constitutive form COX-1 and an inducible form COX-2. COX-2 can be readily induced by cellular stresses, growth factors, cancer promoters, and pro-inflammatory signals [24–30]. Although a few conflicting reports exist, high COX-2 expression has been found in the majority of breast carcinomas, ~ 63%-85% of premalignant-stage breast cancers cases (e.g., ductal carcinoma in situ), and ~ 87% of metastatic breast cancer [24–30]. Induction of COX-2 has also been reported in breast cancer associated fibroblasts that make up the bulk of cancer stroma to promote breast cancer initiation and progression [29]. Among many putative mechanisms by which ω-6 s can modulate the carcinogenic process, COX-2-catalyzed arachidonic acid peroxidation to form PGE2 is the most salient one [15–19]. PGE2 has been shown to be involved in breast cancer growth, invasion, metastasis as well as in the development of chemo-drug resistance [15–19]. A variety of COX-2 inhibitors and ω-3 fatty acid supplements aiming to limit PGE2 formation from COX-2-mediated AA peroxidation have been extensively investigated as a complementary treatment for cancer therapy [31–38]. However, COX-2 inhibitors not only are ineffective in general, but also suffer from critical safety issues in patients such as increased risks of cardiovascular disease and gastrointestinal tract injury [39–42].

Our lab has previously identified both common as well as exclusive free radicals generated from COX-catalyzed peroxidation of AA and dihomo-γ-linolenic acid (DGLA, an intermediate precursor of AA), using a HPLC/ESR/MS combined method along with spin trapping technique [43, 44]. The different structural moiety in DGLA leads to the formation of a distinct byproduct 8-hydroxyoctanoic acid (8-HOA) from COX-2-mediated DGLA peroxidation. More recently, we demonstrated that 8-HOA can serve as a histone deacetylase inhibitor (HDACi) to inhibit growth and metastasis of colon (HCA-7 colony 29 and HT-29) and pancreatic (BxPC-3) cancer cells overexpressing COX-2 [45–49]. Our novel strategy combining genetic knockdown of delta-5-desaturase (D5D), the key enzyme for converting DGLA to AA, with DGLA supplementation has been shown to suppress cancer cell growth and metastasis via not only suppressing PGE2 generation from AA (limited by D5D downregulation), but also by reserving more DGLA to form 8-HOA. Unlike the classic COX-2 inhibition strategy in cancer treatment in which high expression of COX-2 is the problem, we actually now take advantage of high COX-2 expression in cancer cells for the inhibition of cancer growth and metastasis. In addition, our strategy enhances efficacies of many chemo-drugs [45–49].

In the present study, we further extend our strategy to the inhibition of breast cancer growth, migration and invasion. The promoted formation of 8-HOA from COX-2- mediated DGLA peroxidation manipulated by D5D knockdown can significantly inhibit breast cancer cell growth and metastasis, as well as improve the efficacy of 5-fluorouracil, a commonly used chemo-drug to treat breast cancer. The outcomes from our work shed the light on the development of complementary ω-6-based diet care strategies in combination with chemo-drugs for breast cancer treatment.

## Methods

### Cell lines and materials

The human breast cancer cell line MDA-MB 231 (ATCC, catalog # HTB-26) and mouse breast cancer cell line 4 T1 (ATCC, catalog # CRL2539), both with high level of COX-2 expression, were generous gift from Dr. Keith Miskimins (Cancer Biology Research Center, Sanford Research, Sioux Falls, SD). The cells were grown in Dulbecco’s Modified Eagle’s Medium supplemented with 10% fetal bovine serum (Thermo Fisher Scientific, UT, USA). Cells were cultured in an incubator (37 °C) with 5% CO_2_ and a 95% humidified atmosphere. Ethics approval and informed consent are not needed for the use of the cell lines in our study.

DGLA was obtained from Nu-Chek-Prep (MN, USA). 8-HOA, 5-FU, CelLytic™ lysis reagent, and D5D primary antibody produced from rabbit were acquired from Sigma-Aldrich (MO, USA). D5D-targeting siRNA (catalog 4,390,825), negative control siRNA (NC-si) and Lipofectamine™ RNAiMAX transfection reagent were purchased from Life Technologies (NY, USA). GlutaMAX™ Opti-MEM reduced serum medium, Pierce ECL western blot substrates, NE-PER™ nuclear and cytoplasmic extraction reagents were bought from Thermo Fisher Scientific (MA, USA). Annexin V Apoptosis Detection Kit I was obtained from BD Pharmingen™ (NJ, USA). HDAC activity assay kit was purchased from BioVision (CA, USA). COX-2 primary antibody produced in rabbit was acquired from Abcam (MA, USA). γH_2_AX primary antibody was purchased from Bethyl Laboratories (TX, USA). All other primary and secondary antibodies were bought from Cell Signaling (MA, USA). X-ray film was purchased from Phoenix Research Products (NC, USA).

Using BLOCK-IT™ RNAi Designer (http://www.invitrogen.com/rnai), DNA oligos encoding D5D-targeted shRNA were designed with sequence of TGCTGTAATCATCCAGGCCAAGTCCA GTTTTGGCCACTGACTGACTGGACTTGCTGGATGATTA (top strand) and CCTGTAAT CATCCAGCAAGTCCAGTCAGTCAGTCAGTGGCCAAAACTGGACTTGGCCTGGATGATTAC (bottom strand). The oligos were then synthesized and purchased from Integrated DNA Technologies (IA, USA). pcDNA™ 6.2-GW/EmGFP-miR vector was purchased from Invitrogen (NY, USA).

### SiRNA transfection

Breast cancer cells MDA-MB 231 and 4 T1 were seeded at 3.0 × 10^5^ cells per well in a 6-well plate and incubated overnight. After removing cell culture medium, cells in each well were washed with phosphate buffered saline (PBS) and treated with 1.0 mL of transfection mixture containing 10 μL Lipofectamine™ RNAiMAX transfection reagent and D5D siRNA (final concentration 150 nM) diluted in GlutaMAX™ Opti-MEM reduced serum medium. Following 6 h transfection, Dulbecco’s Modified Eagle’s Medium supplemented with 10% fetal bovine serum was to replace the Opti-MEM reduced serum medium. After 48 h incubation, the transfected cells were prepared for western blot evaluation or subjected to further treatments followed by colony formation assay, cell cycle distribution and apoptosis analysis, and GC/MS measurement. Cancer cells transfected with a non-target siRNA were used as negative controls in all described experiments.

### ShRNA transfection

ShRNA D5D transfections of MDA-MB 231 and 4 T1 cells were also conducted, typically for assessment of cancer cell migration and invasion study. It takes more than 48 h to grow cancer cells to 90% confluency for wound healing assay, thus, shRNA transfected cell lines were used instead of siRNA transfected cells to guarantee suppressed D5D activities during the experiment. Briefly, we transfected D5D shRNA into MDA-MB 231 and 4 T1 cells to create stable D5D knockdown cell lines. The DNA oligos encoding D5D-targeted shRNA were cloned into pcDNA™ 6.2-GW/EmGFP-miR vector and transformed into *E. coli*. The shRNA expressed vector was extracted and transfected into cells using X-tremeGENE HP DNA Transfection Reagent (Roche). Stable D5D knockdown MDA-MB 231 and 4 T1 cell lines were selected using blasticidin. The stable D5D knockdown cell lines were used for assessing cancer cell migration and invasion upon different treatments (e.g., DGLA and 5-FU). Cancer cells transfected with negative control shRNA (NC-sh) were used as controls.

### Colony formation assay

Cell growth response upon 8-HOA, DGLA and chemo drug treatment was assessed by colony formation assay as described elsewhere [47–49]. Briefly, wild type-D5D (*wt*-D5D) cells, D5D-knockdown (D5D-*KD)* cells or negative siRNA transfected control (NC-si) cells were seeded at 1000 cells per well into a 6-well plates, and then exposed to 48 h treatment with 8-HOA, DGLA, 5-FU, or their combination. After washing with PBS, the cells were re-incubated with fresh medium for another 10 days, followed by fixing with 10% neutral buffered formalin and staining with 0.05% crystal violet solution. The plates were washed with water and left to dry, then cell colonies in each well were counted using a microcopy. The plate efficiency was calculated as total number of colonies counted in each well divided by total number of cells seeded. Cell survival fraction was calculated as the percentage of plate efficiency from treatment group *vs.* the plate efficiency from vehicle control groups.

### Wound healing assay

Wound healing assay was used to assess cancer cell migration upon treatments of 8-HOA and DGLA. Negative control shRNA transfected (NC-sh) or shRNA transfected D5D-*KD* MDA-MB 231 and 4 T1 cells were seeded 1.0 × 10^6^ cells per well (6-well plate). After the cells reached 90% confluence, a wound was simulated on the cell monolayer by scratching with a sterile pipette tip and each well was then washed with phosphate buffered saline (PBS) to eliminate dislodged cells. The medium was changed to medium with 1.0% fetal bovine serum. The cells were subjected to different treatment (e.g. 8-HOA and DGLA) up to 48 h. The wound area was measured using Image-J software (NIH, Bethesda, MD, USA). The percentages of wound areas were calculated at 24 h and/or 48 h vs. controls (0 h time point) in each group.

### Transwell assay

Transwell migration assays were performed to assess cancer cell migration upon treatments with DGLA and chemo-drugs in transwell chamber with the non-coated membrane (24-well insert, pore size: 8 mm, Corning, Life Sciences). Treated with DGLA or chemo-drugs for 48 h, shRNA transfected D5D-*KD* MDA-MB 231 and 4 T1 cells were trypsinized and counted. 5 × 10^4^ cells from each treatment were plated in the top chamber and incubated overnight to allow the cells to attach. Medium without serum was added to the upper chamber, and the medium containing 10% fetal bovine serum was added in the lower chamber. The cells were fixed in 10% neutral buffered formalin solution for 30 min and stained with 0.05% crystal violet solution for 30 min, and the cells that migrated or invaded through the pores to the lower surface of the inserts were counted under an inverted microscope. For invasion assays, same treatments were also used, except that the transwell inserts were coated with Matrigel.

### Measurement of endogenous 8-HOA in cancer cells

8-HOA (in its derivatized form with pentafluorobenzyl bromide, PFB-Br) generated from D5D-*KD* MDA-MB 231 and 4 T1 cells and negative control cells treated by DGLA were measured via GC/MS as described elsewhere [47–50]. Briefly, 3.0×10^5^ cells were seeded overnight in each well of 6-well plates, transfected with D5D siRNA or its negative control, and received 100 μM of DGLA treatment (in ethanol, final volume < 0.1%) up to 48 h. At each experimental time points, the cells were scratched and collected in 1.0 mL culture medium, and mixed with 500 μL of methanol containing internal standard (hexanoic acid), 50 μL of 1.0 N HCl, and 3.0 mL of dichloromethane. After vortex for 30s, the mixture was centrifuged at 3000 rpm for 4 min and the dichloromethane layer was collected. The samples were subject to the same extraction again, and all organic layers of two extractions were combined and evaporated to dryness. The extracted analytes were reconstituted in 50 μL of 1.0% di-isopropylethylamine in acetonitrile and derivatized with 50 μL of 1.0% PFB-bromide in acetonitrile at 37 °C for 30 min. The acetonitrile was then evaporated to dryness, and the residue was reconstituted in 100 μL dichloromethane for GC/MS analysis.

About 2.0 μL of the sample solution was injected into an Agilent 7890A gas chromatograph. The GC oven temperature (from 60 °C to 300 °C) is programmed at 25 °C/min, while keeping the injector and transfer line at 280 °C. A MS selective detector was used for quantitative analysis with a source temperature of 230 °C. The formation of 8-HOA (in its PFB derivative) was quantified in selected ion monitoring mode for the base peak of 8-HOA-PFB derivative (m/z 181) by comparing it with the base peak of internal standard (hexanoic acid-PFB derivative) using an internal standard curve.

### Cell apoptosis assay

Cell apoptosis of D5D-*KD* MDA-MB 231 and 4 T1 cells as well as their negative controls, upon treatments of DGLA, 5-FU, and the combination, was analyzed using the Annexin V Apoptosis Detection Kit I (BD Pharmingen™, NJ, USA) according to the manufacturer’s instruction [47–49]. Briefly, 3.0×10^5^ cells were seeded overnight in each well of 6-well plates, transfected with D5D siRNA or the negative control, and treated with DGLA, 5-FU or their combination for 48 h. Then the experimental cells were harvested and re-suspended in 1× binding buffer at a concentration of 1.0×10^6^ cells/ml. The cell suspension (~ 100 μl) was then treated with 5.0 μL each of FITC Annexin V and PI solution, gently vortexed, incubated for 15 min at 25 °C in the dark, and finally mixed with 400 μL of 1× binding buffer. The apoptotic cell population was determined on an Accuri C6 flow cytometer within 1 h. 10,000-cell events were counted for each sample. Unstained cells, the cells stained with FITC Annexin V only and PI only, were all used to set up compensation and quadrants. Data was analyzed by FlowJo (TreeStar, Ashland, OR, USA).

### HDAC activity assay

HDAC activity was performed using HDAC activity assay kit according to manufacturer’s instructions. Briefly, after cells directly treated with 8-HOA and D5D-KD along with DGLA, nuclear proteins were extracted with NE-PER™ nuclear and cytoplasmic extraction reagents. Nuclear extracts were incubated with HDAC substrate at 37 °C for 1 h and then lysine developer was added to the mixture and incubated for 30 min at 37 °C. The plate was read at 405 nm on a microplate reader. The HDAC activity in MDA-MB 231 and 4 T1 cells without 8-HOA treatment was set to 100%.

### Western blotting

Western blot was used to assess the expression of D5D, COX-2, γH_2_AX, acetyl-histone H3 (AcH3) as well as proteins involved in apoptosis, migration and invasion in MDA-MB 231 and 4 T1 cells upon treatments [47–49]. The cells seeded in a 6-well plate were transfected with D5D siRNA/shRNA or negative control siRNA/shRNA, and treated with DGLA and/or 5-FU for 48 h. The proteins were extracted from experimental cells and then loaded into 10% SDS-PAGE gels. The gel was ran at a constant current of 30 mA for 1 h followed by protein transferring at a constant voltage of 80 V for 2 h on ice. The membranes were incubated with primary antibodies (1:600 dilution) overnight at 4 °C and horseradish peroxidase-conjugated secondary antibody (1:2000 dilution) for 1 h at room temperature with continuous rocking. The membranes were then incubated in ECL western blot substrates for 1 min, and exposed to X-ray film. Luminescent signals were captured on a Mini-Medical Automatic Film Processor (Imageworks). Image data was analyzed by ImageJ software.

### Statistic Analysis

Statistical analysis was performed on all data using student’s unpaired t-test (two-tailed). A statistically significant difference was considered with a *p* value less than 0.05.

## Results

### 8-HOA inhibits breast cancer cell growth and migration

**Fig. 1:**
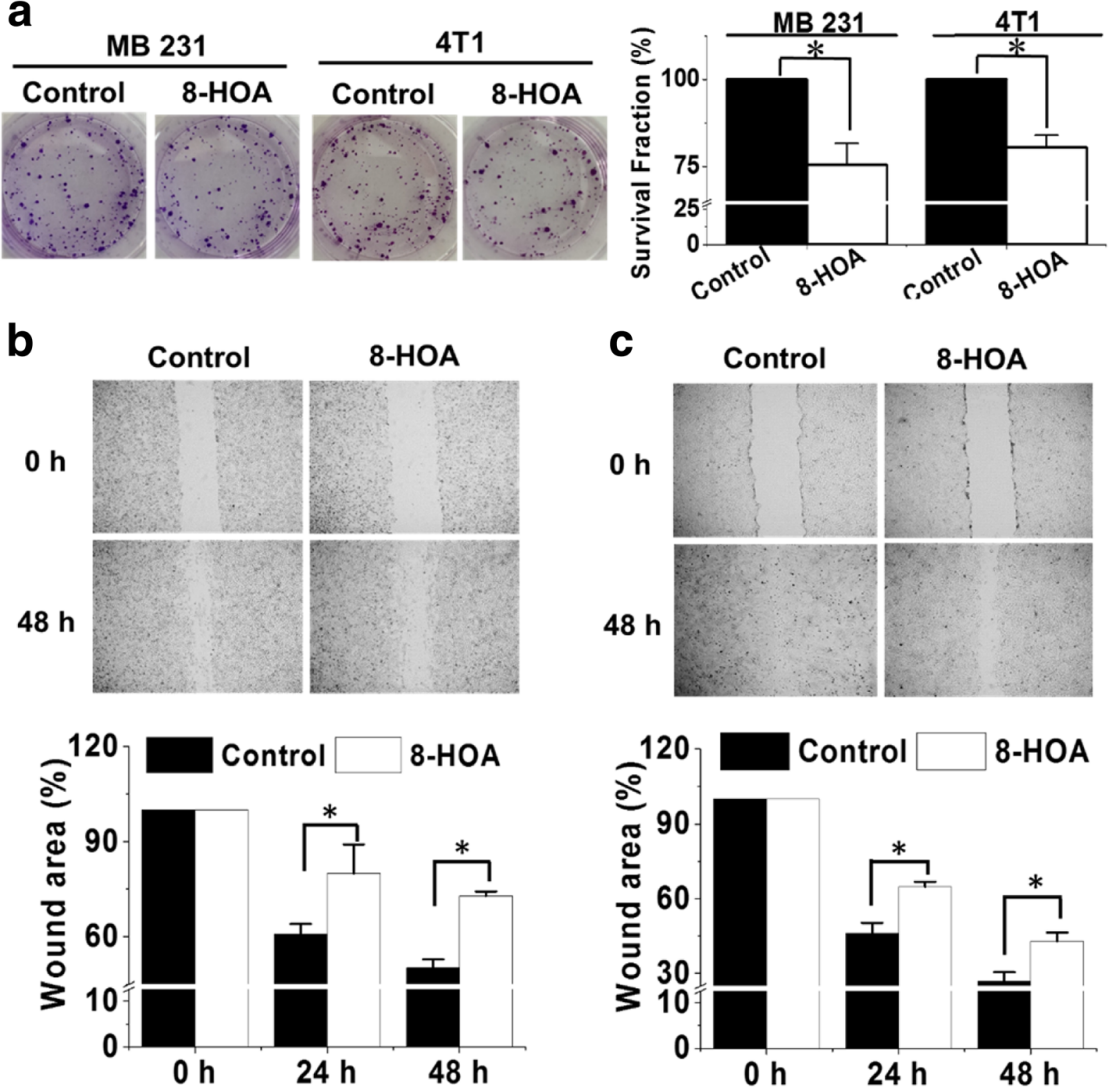
8-HOA inhibits breast cancer cell growth and migration. **a** Colony formation of MDA-MB 231 and 4 T1 cells at 10 days after 8-HOA treatment (1.0 μM for 48 h) and quantification analysis; **b** Wound healing assay and quantification of MDA-MB-231 cells upon 8-HOA treatment (1.0 μM) for 48 h. The MDA-MB 231 cells treated with vehicle were used as control; and **c** Wound healing assay and quantification of 4 T1 cells upon 8-HOA treatment (1.0 μM) for 48 h. The 4 T1 cells treated with vehicle were used as control. Data represent as mean ± standard deviation (*: significant difference with *p* < 0.05 from *n* ≥ 3)

Treatment of 8-HOA (1.0 μM, 48 h) significantly suppressed the colony formation of MDA-MB 231 cells in a reduced surviving fraction of 75.5 ± 6.2% *vs.* 100% in control (no 8-HOA treatment, Fig. 1a, *p* < 0.05). 8-HOA treatment (1.0 μM, 48 h) also greatly suppressed the colony formation of 4 T1 cells (surviving fraction of 80.5 ± 3.6 *vs.* 100% in control, *p* < 0.05).

Direct treatment of 8-HOA can also significantly inhibit cancer cell migration. Using the wound healing assay, MDA-MB 231 cells exhibited 72.8% of the original wound areas upon 8-HOA treatment 48 h *vs.* 50.1% in control (no 8-HOA treatment, Fig. 1b, *p* < 0.05). Similarly, suppressed migration of 4 T1 cells was also observed upon 8-HOA treatment with a wound area at 48 h 42.9% *vs.* 26.9% in control (Fig. 1c, *p* < 0.05).

### D5D-KD promoted 8-HOA formation to inhibit breast cancer cell growth and migration

**Fig. 2:**
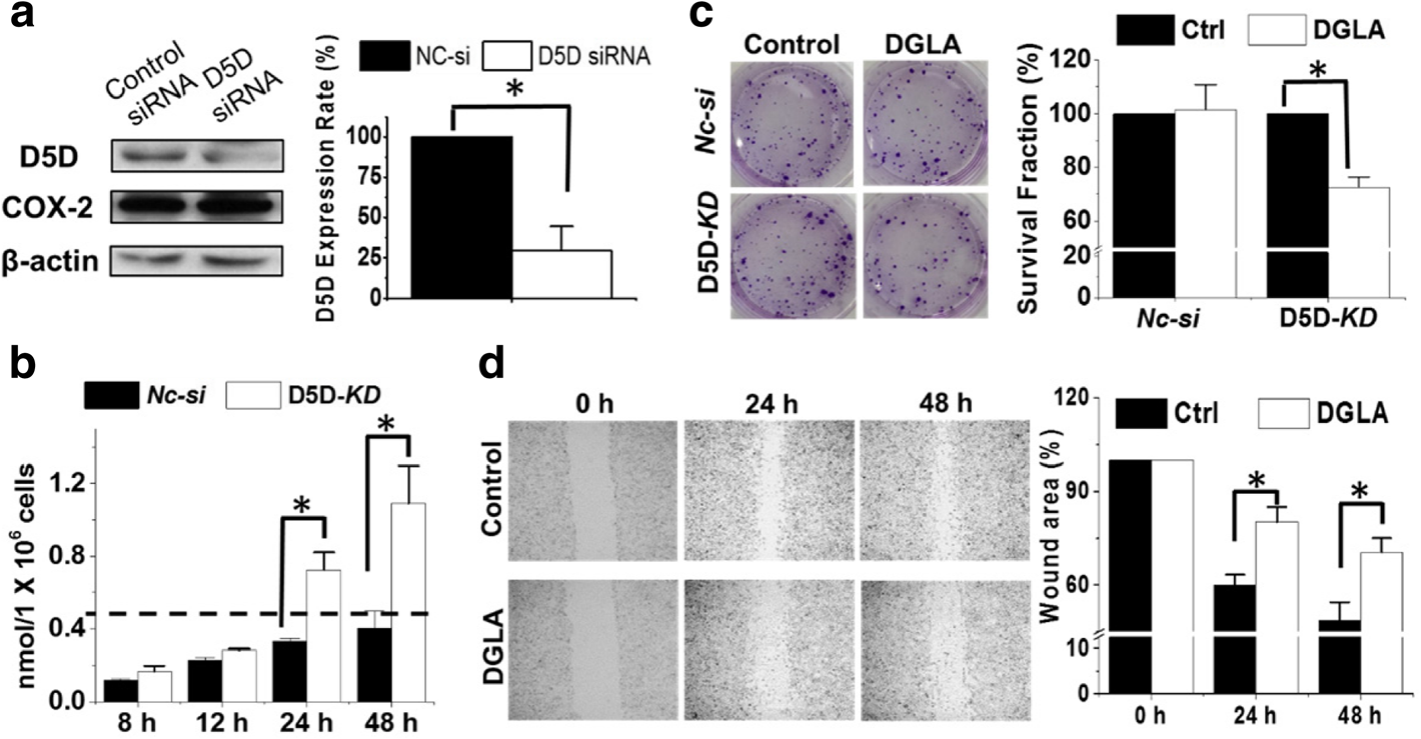
D5D-*KD* and DGLA inhibit MDA-MB 231 cell growth and migration. **a** Western blot and protein expression rate of D5D and COX-2 in MDA-MB 231cells after D5D siRNA transfection (β-actin as loading control). Similar inhibition of D5D (~ 70%) was also observed in shRNA transfected MDA-MB-231 cells; **b** GC/MS quantification of 8-HOA from cell medium containing 1.0 × 10^6^ siRNA D5D-*KD* MDA-MB 231 cells or control siRNA transfected cells after 100 μM DGLA treatment. Similar results were also observed in shRNA transfected cell lines vs. their controls (data not shown); **c** Colony formation of D5D-*KD* MDA-MB231 or control siRNA transfected cells 10 days after DGLA treatment (100 μM for 48 h); and **d** Wound healing assays and quantification of wound area of D5D-*KD* MDA-MB 231 cells upon DGLA (100 μM, 48 h) treatment vs. controls (without DGLA). Data represent as mean ± standard deviation (*: significant difference with *p* < 0.05 from *n* ≥ 3)

When MDA-MB 231 cells were transfected with D5D-targeted siRNA and treated with 100 μM DGLA, endogenous 8-HOA accumulated and reached a threshold and physiological level ≥ 0.5 μM (nmol/1 × 10^6^ cells/mL) [47–49] at 24 h and 48 h (Fig. 2a and b). By comparison, in negative control siRNA transfected cells (Nc-si) treated with DGLA, the 8-HOA formation never reach 0.5 μM threshold level during 48 h incubation (Fig. 2b). In addition, when COX-2 was knocked down in cancer cells, only trace level of 8-HOA was detected (Additional file 1: Figure S1), indicating the formation of 8-HOA is COX-2 dependent. In D5D-*KD* MDA-MB 231 cells, with the promoted 8-HOA formation, DGLA supplementation (100 μM) significantly suppressed cancer cell growth with surviving fraction of ~ 72.5 ± 3.9% vs. 100% in control (no DGLA, Fig. 2c, *p* < 0.05). In contrast, in control *NC-si* cells, DGLA supplementation could not inhibit cancer cell growth since 8-HOA formation never reached the threshold level due to the putative conversion of DGLA to the downstream fatty acid AA. These observations suggested that D5D-*KD* can promote 8-HOA formation from COX-catalyzed DGLA peroxidation, thus inhibiting the growth of human breast cancer cells that overexpress COX-2.

**Fig. 3:**
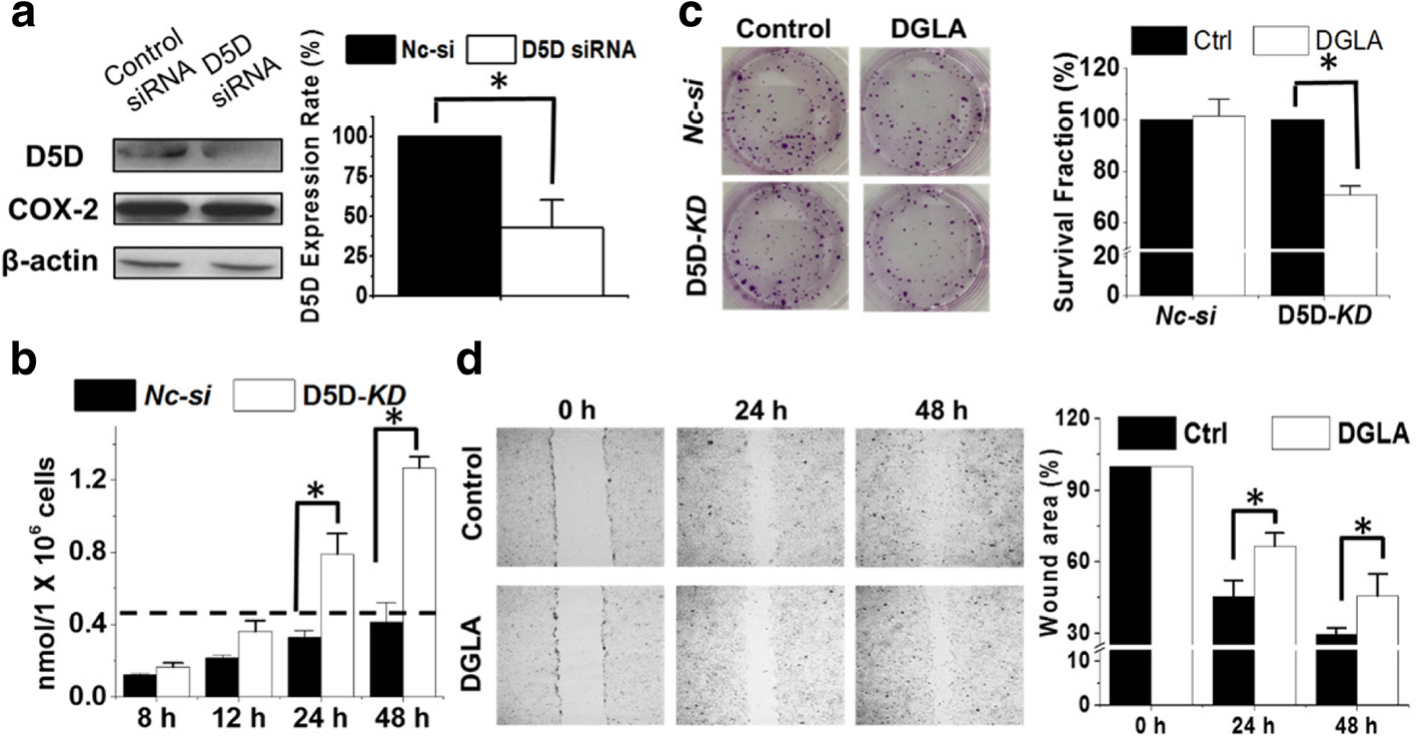
D5D-*KD* and DGLA inhibit 4 T1 cells growth and migration. **a** Western blot and protein expression rate of D5D and COX-2 in 4 T1 cells after D5D siRNA transfection (β-actin as loading control). Similar inhibition of D5D was also observed in shRNA transfected 4 T1 cells; **b** GC/MS quantification of 8-HOA from cell medium containing 1.0 × 10^6^ D5D-*KD* 4 T1 cells or control siRNA transfected cells after 100 μM DGLA treatment, Similar results were also observed in shRNA transfected cell lines vs. their controls (data not shown); **c** Colony formation of D5D-*KD* 4 T1 or control siRNA transfected cells at 10 days after DGLA treatment (100 μM for 48 h); and **d** Wound healing assays and quantification of wound area of D5D-*KD* 4 T1 cells upon DGLA (100 μM, 48 h) treatment vs. controls (without DGLA). Data represent as mean ± standard deviation (*: significant difference with *p* < 0.05 from *n* ≥ 3)

When 4 T1 cells were transfected with D5D-targeted siRNA and treated with 100 μM DGLA, 8-HOA also accumulated and reached a threshold level of ≥ 0.5 μM [47–49] at 24 h and 48 h (Fig. 3a and b). However, the formation of 8-HOA in negative control siRNA transfected cells never reach 0.5 μM (Fig. 3b). DGLA supplementation (100 μM) in D5D-*KD* 4 T1 cells also significantly suppressed cancer cell growth generating a surviving fraction of 70.8 ± 3.6% vs. 100% in control (no DGLA treatment, Fig. 3c, *p* < 0.05). Again in *NC-si* 4 T1 cells, DGLA supplementation did not inhibit cancer cell growth, confirming that the growth inhibitory effect of DGLA is derived from increased 8-HOA from the D5D-*KD* strategy.

Endogenous 8-HOA in D5D-*KD* cells (via shRNA transfection) after DGLA supplementation can also greatly inhibit cancer cell migration. There was significantly suppressed migration in D5D-*KD* MDA-MB 231 cells upon DGLA treatment 48 h with a wound area of 70.5% vs. 48.8% in control (Fig. 2d, *p* < 0.05), and inhibited migration of D5D-*KD* 4 T1 cells upon DGLA treatment 48 h with a wound area of 45.5% vs. 29.6% in control (Fig. 3d, *p* < 0.05). Note, no differences in wound areas were observed upon 48 h DGLA treatment for either NC-sh MDA-MB 231 or NC-sh 4 T1 cell lines (Additional file 2: Figure S2).

### D5D-KD (siRNA transfection) enhances the efficacy of 5-FU on breast cancer cell growth

**Fig. 4:**
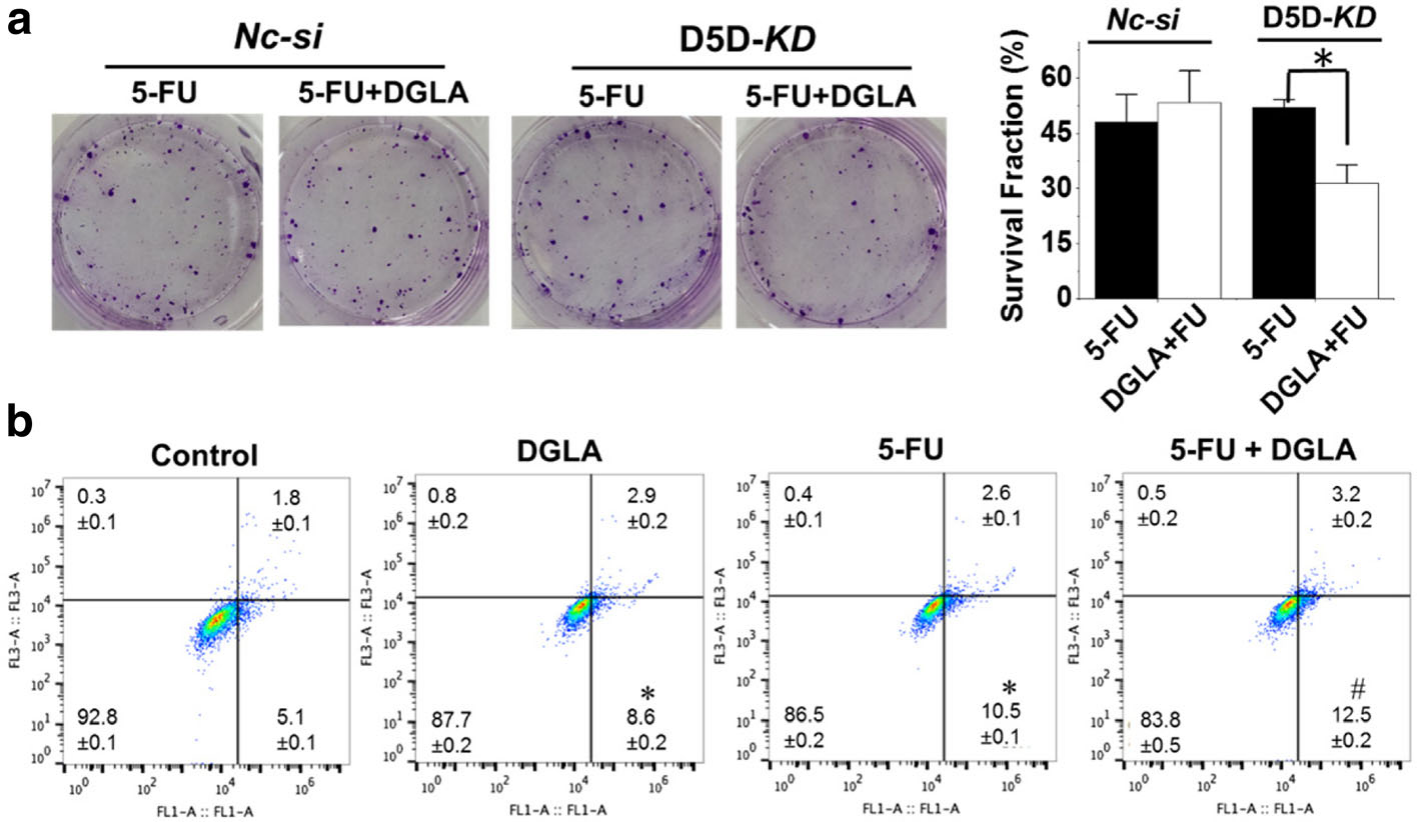
D5D-*KD* and DGLA supplementation enhance the efficacy of 5-FU on growth of MDA-MB 231 cells. **a** Colony formation assay of D5D-*KD* MDA-MB 231 cells or control siRNA transfected cells at 10 days with treatment of 5-FU (10 μM) or 5-FU + DGLA (100 μM) for 48 h. Note, D5D-*KD* and NC-si control cells without DGLA and 5-FU treatment (shown in Fig. 2) were used to calculate survival fractions; and **b** Cell apoptosis was examined via flow cytometry after D5D-*KD* MDA-MB 231 cells were treated with DGLA (100 μM), 5-FU (10 μM), or 5-FU + DGLA for 48 h, followed by Annexin V-FITC/PI double staining. Data represent as mean ± standard deviation (*: significant difference vs. control with *p* < 0.05; #: significant difference vs. 5-FU group with *p* < 0.05 from *n* ≥ 3)

Co-treatment of DGLA (100 μM) and 5-FU (10 μM) in D5D-*KD* MDA-MB 231 cells enhanced cell growth inhibition, and resuted in a surviving fraction 31.5 ± 5.1% vs. 52.1% ± 2.0% in 5-FU treatment alone (Fig. 4a), while in *NC-si* cells, the co-treatment did not lead to any improvement compared to 5-FU treament alone. Concurrent DGLA treatment (100 μM) also promoted 5-FU-induced apoptosis in D5D*-KD* MDA*-*MB 231 cells. Flow cytometric analysis revealed an increased early apoptotic cell population of 12.5% ± 0.2% (annexin V^+^/PI^−^ staining) compared to that in 5-FU only group (10.5% ± 0.1%, Fig. 4b).

**Fig. 5:**
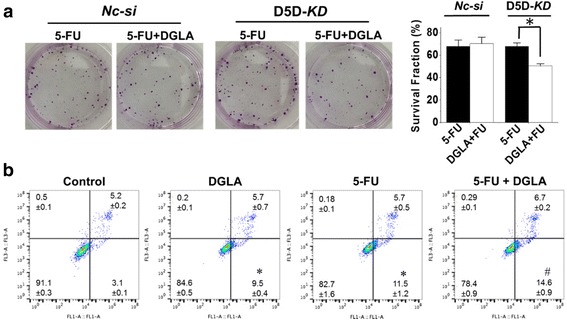
D5D-*KD* and DGLA supplementation enhance the efficacy of 5-FU on growth of 4 T1 cells. **a** Colony formation assay of D5D-*KD* 4 T1 cells or control siRNA transfected cells at 10 days with treatment of 5-FU (20 μM) or 5-FU + DGLA (100 μM) for 48 h. Note, D5D-*KD* and NC-si control cells without DGLA and 5-FU treatment (shown in Fig. 3) were used to calculate survival fractions; and **b** Cell apoptosis was examined via flow cytometry after D5D-*KD* 4 T1 cells were treated with DGLA (100 μM), 5-FU (20 μM), or 5-FU + DGLA for 48 h, followed by Annexin V-FITC/PI double staining Data represent as mean ± standard deviation (*: significant difference vs. control with *p* < 0.05; and #: significant difference vs. 5-FU group with *p* < 0.05 from *n* ≥ 3)

D5D-*KD* along with DGLA supplementation also enhanced the efficacy of 5-FU on the growth inhibition of 4 T1 cells. For example, co-treatment of DGLA (100 μM) and 5-FU (20 μM) in D5D-*KD* 4 T1 cells enhanced cell growth inhibition with a surviving fraction 50.3 ± 1.9% vs. 67.7% ± 3.4% in 5-FU treatment alone (Fig. 5a), while in *NC-si* cells the co-treatment did not improve the growth inhibition effect compared to 5-FU treament alone. DGLA supplementation also promoted 5-FU-induced apoptosis of D5D-*KD* 4 T1 cells (early apoptotic cell population of 14.6% ± 0.9%, Fig. 5b) vs. 5-FU alone (11.5% ± 1.2%, Fig. 5b).

### D5D-KD (shRNA transfection) enhances the efficacy of 5-FU on breast cancer cell migration

**Fig. 6:**
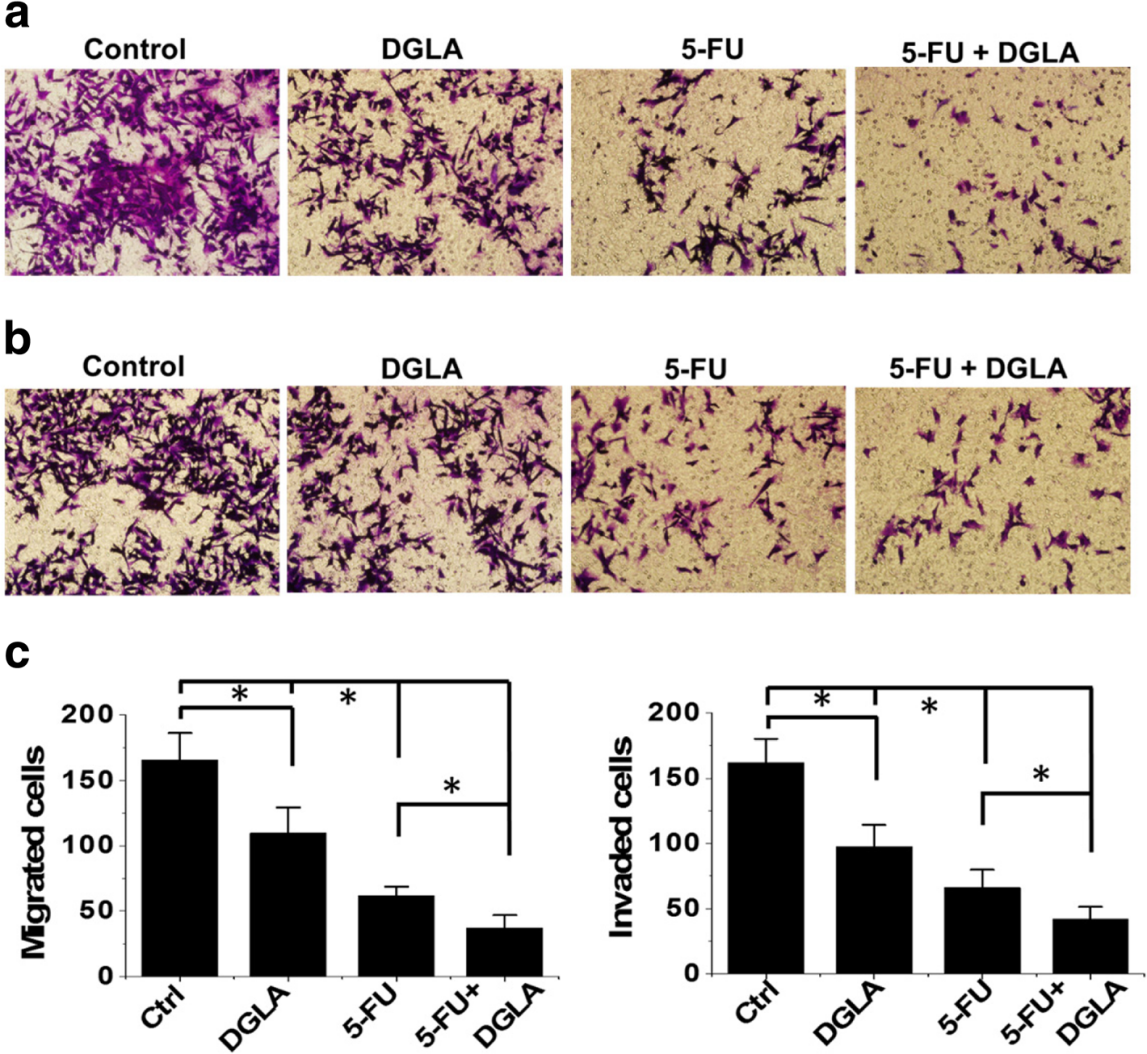
Efficacy of 5-FU on cell migration and invasion in MDA-MB 231 cells was enhanced by D5D-*KD* (via shRNA) and DGLA treatment. **a** Transwell migration assay of D5D-*KD* MDA-MB 231 cells upon treatment of DGLA (100 μM), 5-FU (20 μM) alone or 5-FU + DGLA. The D5D-*KD* cells without fatty acid and drug treatment were used as controls; **b** Transwell invasion assay of D5D-*KD* MDA-MB 231 cells upon treatment of DGLA (100 μM), 5-FU (20 μM) alone or 5-FU + DGLA. The D5D-*KD* cells without fatty acid and drug treatment were used as controls; and **c** Quantification of transwell migration and invasion assay of D5D-*KD* MDA-MB 231 cells. Data represent as mean ± standard deviation (*: significant difference with *p* < 0.05 from *n* ≥ 3)

DGLA treatment also enhanced the inhibition effects of 5-FU on the migration of D5D-*KD* MDA-MB 231 cells. Upon co-treatment of DGLA and 5-FU there were approximately 40% less cells that migrated compared to treatment with 5-FU alone (37 cells migrated after co-treatment of DGLA and 5-FU vs. 62 cells after 5-FU treatment, Fig. 6a). Invasion by D5D-*KD* MDA-MB 231 cells was also further inhibited by the co-treatment of DGLA and 5-FU. The number of cells that accomplished invasion upon co-treatment of DGLA and 5-FU was 36% less than treatment with 5-FU alone (number of cells that invaded was 42 for co-treatment vs. 66 for 5-FU, Fig. 6b and c).

**Fig. 7:**
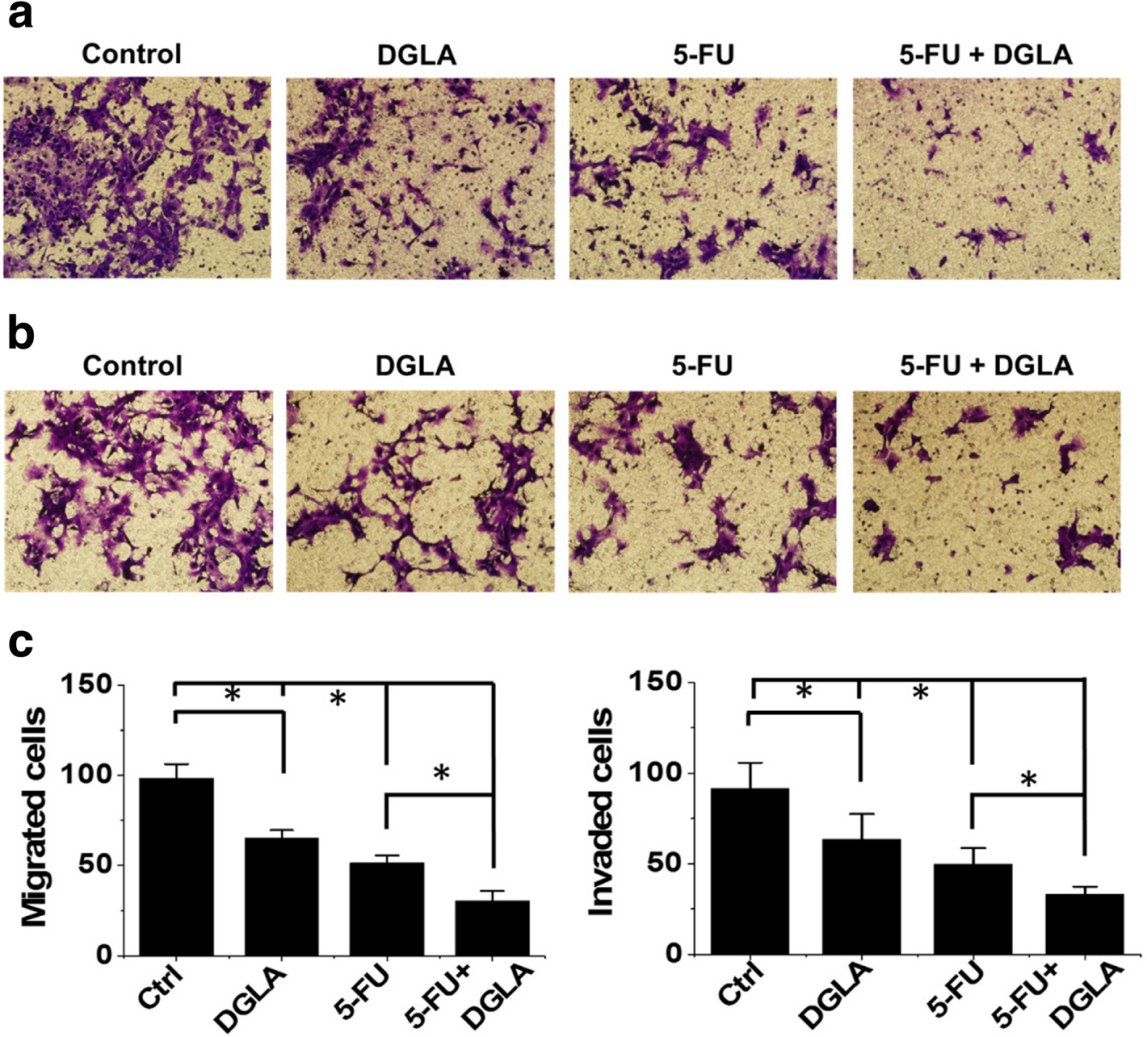
Efficacy of 5-FU on cell migration and invasion in 4 T1 cells was enhanced by D5D-*KD* (via shRNA) and DGLA treatment. **a** Transwell migration assay of D5D-*KD* 4 T1 cells upon treatment of DGLA (100 μM), 5-FU (50 μM) alone or 5-FU + DGLA. The D5D-*KD* cells without fatty acid and drug treatment were used as controls; **b** Transwell invasion assay of D5D-*KD* 4 T1 cells upon treatment of DGLA (100 μM), 5-FU (50 μM) alone or 5-FU + DGLA. The D5D-*KD* cells without fatty acid and drug treatment were used as controls; and **c** Quantification of transwell migration and invasion assay of D5D-*KD* 4 T1 cells. Data represent as mean ± standard deviation (*: significant difference with *p* < 0.05 from *n* ≥ 3)

D5D-*KD* and DGLA treatment also improved the efficacy of 5-FU on the migration of 4 T1 cells. The number of migrated cells upon co-treatment with DGLA and 5-FU was 41% less when compared to treatment with 5-FU alone (30 cells migrated after co-treatment with DGLA and 5-FU vs. 51 cells after 5-FU only, Fig. 7a). The transwell invasion assays showed that co-treatment of DGLA with 5-FU further inhibited cell invasion compared to 5-FU alone in D5D-*KD* 4 T1 cells. The number of cells that invaded upon co-treatment with DGLA and 5-FU was 34% less than treatment with 5-FU (33 cells invaded after co-treatment vs. ~ 50 cells after 5-FU alone, Fig. 7b and c).

### Mechanism of anti-cancer effect from D5D-*KD*, DGLA along with 5-FU in cancer cells

**Fig. 8:**
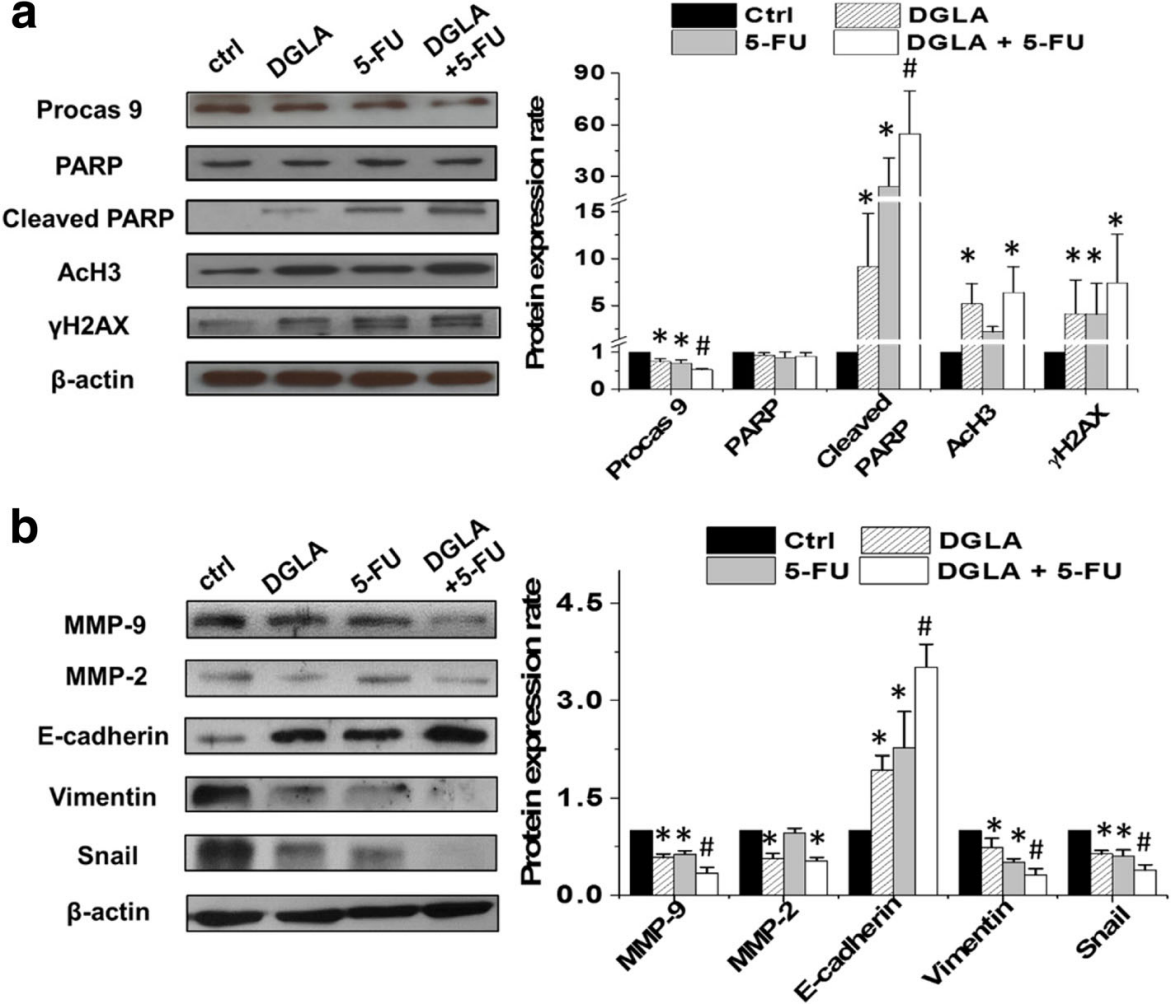
Mechanism of anti-cancer effect from D5D knockdown, DGLA supplementation along with 5-FU in MDA-MB 231 cells. **a** Western blot of procaspase 9, PARP, cleaved PARP, acetyl histone H3, and γH2AX from D5D-*KD* MDA-MB 231 cells treated with 5-FU (20 μM), DGLA (100 μM) and 5-FU + DGLA at 48 h. Protein expression rate was normalized using β-actin as a loading control; and **b** Western blot of MMP-2, MMP-9, E-cadherin, vimentin and snail from D5D-*KD* MDA-MB 231 cells treated with 5-FU (20 μM), DGLA (100 μM) and 5-FU + DGLA at 48 h. Protein expression rate was normalized using β-actin as a loading control. Data represent as mean ± standard deviation (*: significant difference vs. control with *p* < 0.05; and #: significant difference vs. 5-FU group with *p* < 0.05 from *n* ≥ 3)

Consistent with the results demonstrated in colon and pancreatic cancer cells [46–49], both direct 8-HOA treatment and endogenous 8-HOA resulting from D5D-*KD* and DGLA treatment in MDA-MB 231 cells upregulated AcH3 (substrate of histone deacetylase or HDAC) and γH_2_AX (DNA damage marker, Fig. 8a), indicating that 8-HOA can suppress cancer cell growth by inhibiting HDAC and inducing DNA damage.

5-FU inhibited cancer cell growth by inducing DNA damage and cell apoptosis, which was evident by activation of procaspase 9, cleavage of PARP and an increase in the DNA damage marker γH_2_AX (Fig. 8a), consistent with other reports [51, 52]. When the cells were co-treated with DGLA (100 μM) and 5-FU (20 μM), procaspase 9 was more significantly reduced. An increased level of cleaved PARP as well as a slightly increased level of γH_2_AX were observed compared to 5-FU treatment alone in D5D-*KD* MDA-MB 231 cells (Fig. 8a). Note, no change in AcH3 level was observed upon 5-FU treatment alone.

5-FU also inhibited cancer cell migration and invasion by downregulating the expression of MMP-9, mesenchymal marker vimentin and EMT-inducing transcription factor snail and simultaneously upregulating the expression of epithelial marker E-cadherin (Fig. 8b) [53–57]. Decreased expression of MMP-9, vimentin, snail and increased expression of E-cadherin were also observed when D5D-*KD* MDA-MB 231 cells were co-treated with DGLA and 5-FU.

**Fig. 9:**
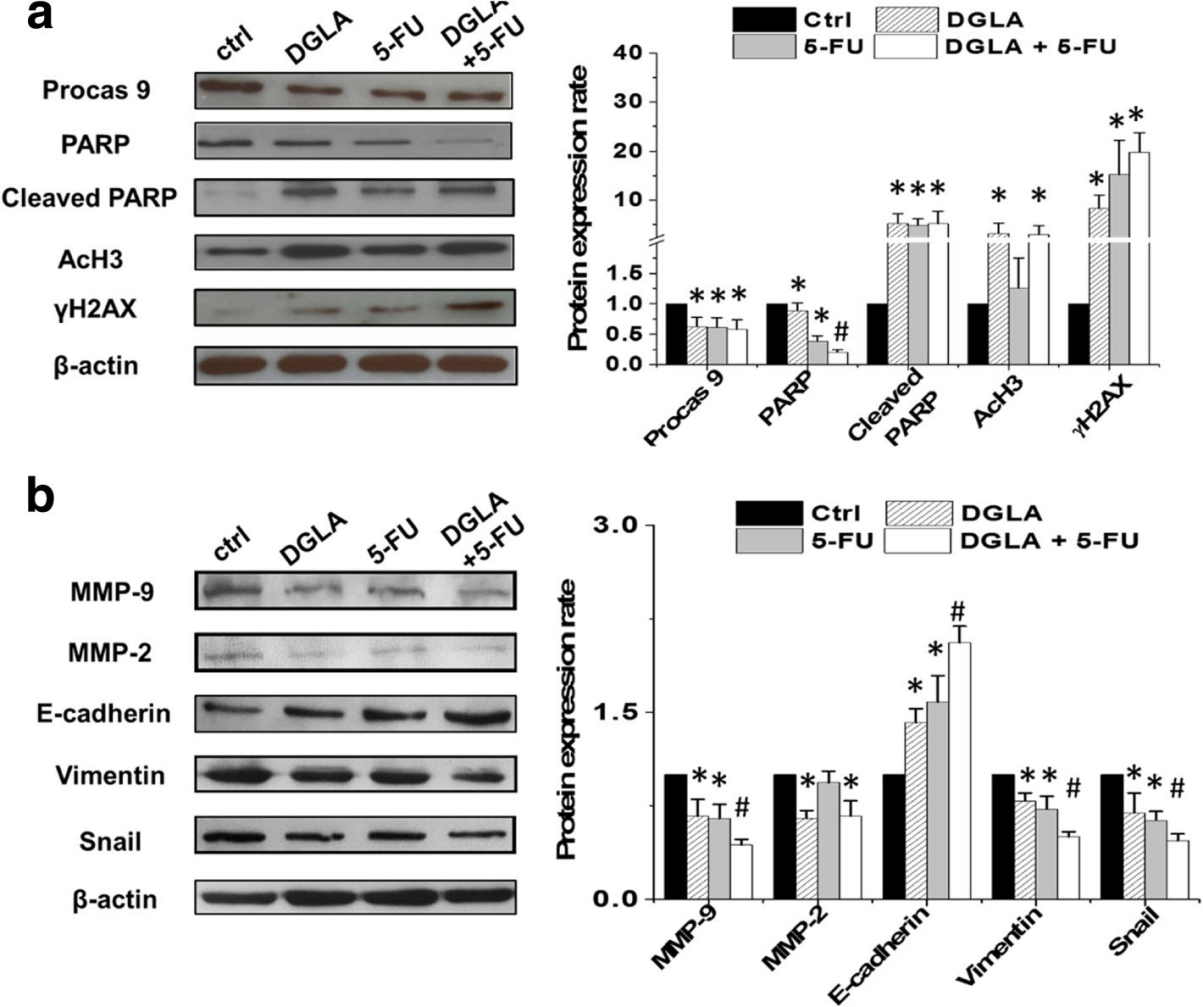
Mechanism of anti-cancer effect from D5D knockdown, DGLA supplementation along with 5-FU in 4 T1 cells. **a** Western blot of procaspase 9, PARP, cleaved PARP, acetyl histone H3, and γH2AX from D5D-*KD* 4 T1 cells treated with 5-FU (50 μM), DGLA (100 μM) and 5-FU + DGLA at 48 h. Protein expression rate was normalized using β-actin as a loading control; and **b** Western blot of MMP-2, MMP-9, E-cadherin, vimentin and snail from D5D-*KD* 4 T1 cells treated with 5-FU (50 μM), DGLA (100 μM) and 5-FU + DGLA at 48 h. Protein expression rate was normalized using β-actin as a loading control. Data represent as mean ± standard deviation (*: significant difference vs. control with *p* < 0.05; and #: significant difference vs. 5-FU group with *p* < 0.05 from *n* ≥ 3)

We also tested the possible anti-cancer mechanisms of our strategy in D5D-*KD* 4 T1 cells. Promoted 8-HOA formation from D5D-KD and DGLA treatment in 4 T1 cells could upregulate AcH3 and γH2AX level (Fig. 9a). The co-treatment of DGLA and 5-FU in D5D-*KD* 4 T1 cells led to significantly less PARP compared to 5-FU treatment alone (Fig. 9a). Treatment with 5-FU alone also decreased expression of MMP-9, vimentin and snail as well as increased expression of E-cadherin in D5D-*KD* 4 T1 cells (Fig. 9b). When co-treated with DGLA and 5-FU, further reduction in expressions of MMP-9, vimentin and snail as well as increased expression of E-cadherin were observed in D5D-*KD* 4 T1 cells compared to the cells with 5-FU treatment alone.

## Discussion

Our previous work demonstrated that the distinct byproduct 8-HOA could be generated from COX-catalyzed peroxidation of DGLA. Further studies showed that 8-HOA can inhibit colon and pancreatic cancer cell growth and metastasis, via inhibiting HDAC and inducing DNA damage [46–49]. We thus proposed that the commonly high COX expression in cancer cells can be taken advantage by inhibiting D5D to promote 8-HOA formation and thus DGLA’s anti-cancer activity. Unlike the classic COX-2 inhibition strategy in cancer treatment where overexpressed COX-2 is the problem, high COX-2 in our new strategy is no longer the problem but instead a benefit to kill cancer cells. Here we demonstrated that D5D knockdown can enhance COX-2 mediated DGLA peroxidation and then promote 8-HOA formation to a threshold level (≥ 0.5 μM), thereby leading to the inhibition of growth and migration in breast cancer cells. Our strategy also greatly enhanced the efficacies of a chemotherapeutic drug (5-FU) in breast cancer.

We observed that direct treatment of 8-HOA at 1.0 μM significantly suppressed the colony formation and migration of MDA-MB 231 and 4 T1 cells (Fig. 1). Here we tested the 8-HOA at 1.0 μM because this is the physiologically relevant concentration of 8-HOA that was detected in our experiment (Figs. 2 and 3). Note, the inhibitory effects from 8-HOA observed in our experiments were only from a single dose treatment. We have recently finished an animal experiments in which we observed that 4-week supplement of DGLA to mice bearing xenograft tumors led to continuous generation and accumulation of 8-HOA, resulting in significant inhibition of tumor growth.

We proposed and demonstrated that there is a threshold level of 8-HOA that is required for eliciting DGLA’s anti-cancer effects [46–49]. For example, 100 μM of DGLA treatment in *D5D-KD* MDA-MB 231 and *D5D-KD* 4 T1 cells resulted in endogenous 8-HOA ≥ 0.5 μM in both of cell lines most time points for 48 h DGLA treatment, which then inhibited their colony formation (Figs. 2-3). In comparison, in both *NC-si* MDA-MB-231 and *NC-si* 4 T1 cells, endogenous 8-HOA never reached 0.5 μM during 48 h DGLA treatment (Figs. 2-3), and is unable to be continually accumulated due to limited free DGLA at 48 h (Additional file 3: Table S1). Therefore no growth inhibitory effect was observed in both of the *NC-si* cells. These observations together suggested that a threshold level of endogenous 8-HOA between 0.5-1.0 μM is required for eliciting anti-cancer activities.

**Fig. 10:**
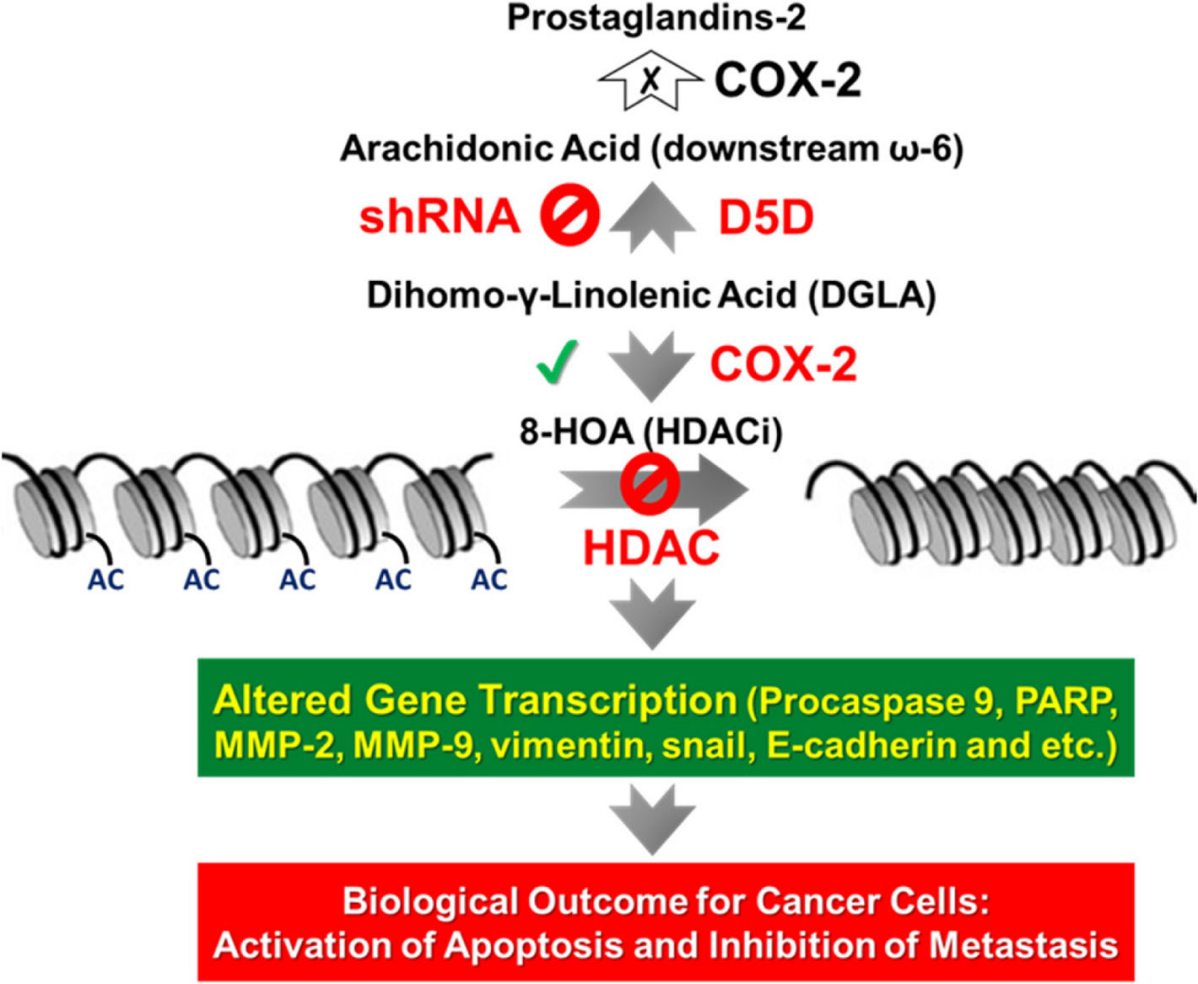
Our proposed novel strategy to inhibit breast cancer growth, migration and invasion

We observed that D5D-*KD* and DGLA supplement promoted formation of 8-HOA which inhibits HDAC, evidence by detected higher level of acetyl histone H3, a substrate of HDAC (Figs. 8-9). Consistently, HDAC activity assay also showed that about 40%-50% of HDAC activity was inhibited in 4 T1 and MDA-MB-231 cells upon either direct treatment of 8-HOA or promoted 8-HOA formation from D5D-*KD* along with 100 μM DGLA treatment (Additional file 4: Figure S3). As HDAC catalyzes histone deacetylation, an important regulatory mechanism for DNA expression, inhibition of HDAC can induce DNA damage and inhibit cancer cell growth and migration. Our study demonstrated that D5D-*KD* along with DGLA treatment can promote formation of 8-HOA which serves as an HDACi to induce DNA damage, and consequently inhibit cancer cell growth and migration, via regulating key signaling proteins, e.g., MMPs-2, 9, e-cadherin, etc, Fig. 10).

5-FU is one of the most commonly used first-line chemo-drug for many types of cancer, including breast cancer [58]. It acts as a thymidylate synthase inhibitor and interferes with DNA synthesis to inhibit cancer cell growth [59–62]. However, many breast cancer cells/tumors are resistant to 5-FU which is a major obstacle for successful cancer chemotherapy [1, 5, 63, 64]. Our study demonstrated that the D5D-*KD* and DGLA supplementation strategy could effectively enhance the efficacy of 5-FU on suppressing cancer growth, migration and invasion [46–49], due to their distinct mechanism to induce apoptosis and DNA damage. Thus, the combined approaches show great potential to be used as a complementary therapy to improve efficacies of many chemo-drugs.

In this study, besides a commonly used human breast cancer cell line MDA-MB 231, we also tested a mouse breast cancer cell line 4 T1 which is an excellent cell line for creating a breast tumor xenograft model in cancer research due to its high potential for growth and metastasis [65, 66]. We have already established a mouse xenograft tumor model using 4 T1 cells for metastasis study [66], which will be continually used in our research to investigate the effect of our strategy on breast cancer growth and metastasis.

Delivery of therapeutic siRNA in cancer therapy has always been challenging. To overcome this problem, RNA nanoparticles have emerged recently as a new platform for in vivo delivery of siRNA and miRNA [67–69]. We are now working to develop thermodynamically and chemically stable RNA nanoparticles harboring D5D siRNA and a cancer targeting ligand for specific delivery of D5D siRNA to cancer cells and tumors. These novel RNA nanoparticles can specifically target cancer with little to no accumulation in healthy tissues, highlighting the benefits of translating RNA nanoparticles for cancer therapy with enhanced targeting efficiency and reduced side effects [67–69].

We have demonstrated that 8-HOA could directly inhibit the growth of cancer cells, not only in cancer cell lines overexpressing COX-2, but also in COX-2 deficient cancer cell lines [46, 47]. Taking into account the fact that many types of tumors commonly overexpress COX-2, we propose that our strategy can also kill low or deficient COX-2 cancer cells in a paracrine-like manner as they surrounded by many other cancer cells (overexpressing COX-2) that produce enough 8-HOA. In addition, our new concept is also supported by a recent study from Dr. Bissonnette’s lab in which COX-2 expression can be induced in a xenograft tumor model (HCT 116, COX-2 deficient colon cancer cell line), particularly in stroma [70].

## Conclusions

In conclusion, the data presented in this study and our previous studies have demonstrated that D5D knockdown is an effective strategy to promote 8-HOA formation from COX-catalyzed DGLA peroxidation, which serves as a HDAC inhibitor to induce DNA damage, activate cell apoptosis and inhibits cancer cell growth and migration. Compared to the classic COX inhibition strategy in cancer treatment, our novel strategy will result in dual inhibitory effects on cancer, which not only limits deleterious PGE2 formation from arachidonic acid (the common objective of COX-2 inhibitor), but also promotes formation of 8-HOA via capitalizing on high COX-2 expression in cancer as well as the abundance of ω-6 s in the daily diet. Thus, our strategy will lead to a better therapeutic outcome with less side effects to treat cancers, including breast cancer.

## Additional files

Additional file 1: **Figure S1.** GC/MS quantification of 8-HOA from *Nc-si*, D5D-*KD* and D5D/COX-2 double-*KD* MDA-MB 231/4 T1 cells after DGLA treatment. (DOCX 71 kb)

Additional file 2: **Figure S2.** Wound healing assays of *NC-sh* MDA-MB-231 and *NC-sh* 4 T1 cells upon DGLA treatment vs. controls. (DOCX 827 kb)

Additional file 3: **Table S1.** LC/MS quantification of DGLA and AA from *Nc-si* MB 231, D5D-*KD* MB 231, *Nc-si* 4 T1 or D5D-*KD* 4 T1 cells after DGLA treatment. (DOCX 17 kb)

Additional file 4: **Figure S3.** HDAC activity assay of D5D-*KD* 4 T1 and D5D-*KD* MDA-MB-231 cells treated with vehicle or DGLA. (DOCX 36 kb)

## Abbreviations

5-FU: 5-flurouracil
8-HOA: 8-hydroxyoctanoic acid.
AA: Arachidonic acid
COX: Cyclooxygenase
D5D: Delta-5-desaturase
D5D-KD: Delta-5-desaturase knockdown
DGLA: Dihomo-γ-linolenic acid
HDAC: Histone deacetylase
HDACi: Histone deacetylase inhibitor
NC-sh: Negative control shRNA transfected
NC-si: Negative control siRNA transfected
