# Supplementary material for: Knockdown delta-5-desaturase in breast cancer cells that overexpress COX-2 results in inhibition of growth, migration and invasion via a dihomo-γ-linolenic acid peroxidation dependent mechanism
Source: BMC Cancer. 2018 Mar 27;18:330. doi: 10.1186/s12885-018-4250-8 (PMC5870477; doi:10.1186/s12885-018-4250-8)
Supplement: Supplementary file 1 — Figure S1. GC/MS quantification of 8-HOA from Nc-si, D5D-KD and D5D/COX-2 double-KD MDA-MB 231/4 T1 cells after DGLA treatment. (DOCX 71 kb) [file 12885_2018_4250_MOESM1_ESM.docx]

**additional file 1**

**Methods**

**D5D/COX-2 double knockdown**

MDA-MB 231 and 4T1 were seeded at 3.0 ×10^5^ cells per well in a 6-well plate and incubated overnight. For COX-2 knockdown only, cells in each well were washed with PBS and treated with 1.0 mL of transfection mixture containing 20 µL Lipofectamine™ RNAiMAX transfection reagent, 150 nM of D5D siRNA and 150 nM COX-2 siRNA (Life Technologies) diluted in GlutaMAX™ Opti-MEM reduced serum medium. Following 6 hr transfection, the Opti-MEM reduced serum medium was replaced with Dulbecco’s Modified Eagle’s Medium supplemented with 10% fetal bovine serum. After 48 hours incubation, the transfected cells were ready for further treatments.

**Measurement of endogenous 8-HOA in cancer cells**

8-HOA produced from *Nc-si*, D5D-*KD* and D5D/COX-2 double-*KD* MDA-MB 231 and 4T1 cells were quantified via GC/MS analysis as described in the main text.

**Figure Legend**

**Supplement Figure 1.** **A.** GC/MS quantification of 8-HOA from cell medium containing 1.0×10^6^ *Nc-si*, D5D-*KD* and D5D/COX-2 double-*KD* MDA-MB 231 cells after 100 μM DGLA treatment; **B.** GC/MS quantification of 8-HOA from cell medium containing 1.0×10^6^ *Nc-si*, D5D-*KD* and D5D/COX-2 double-*KD* 4T1 cells after 100 μM DGLA treatment. Data represent as mean ± standard deviation. (*: significant difference vs. control with p < 0.05, from n ≥ 3).

**
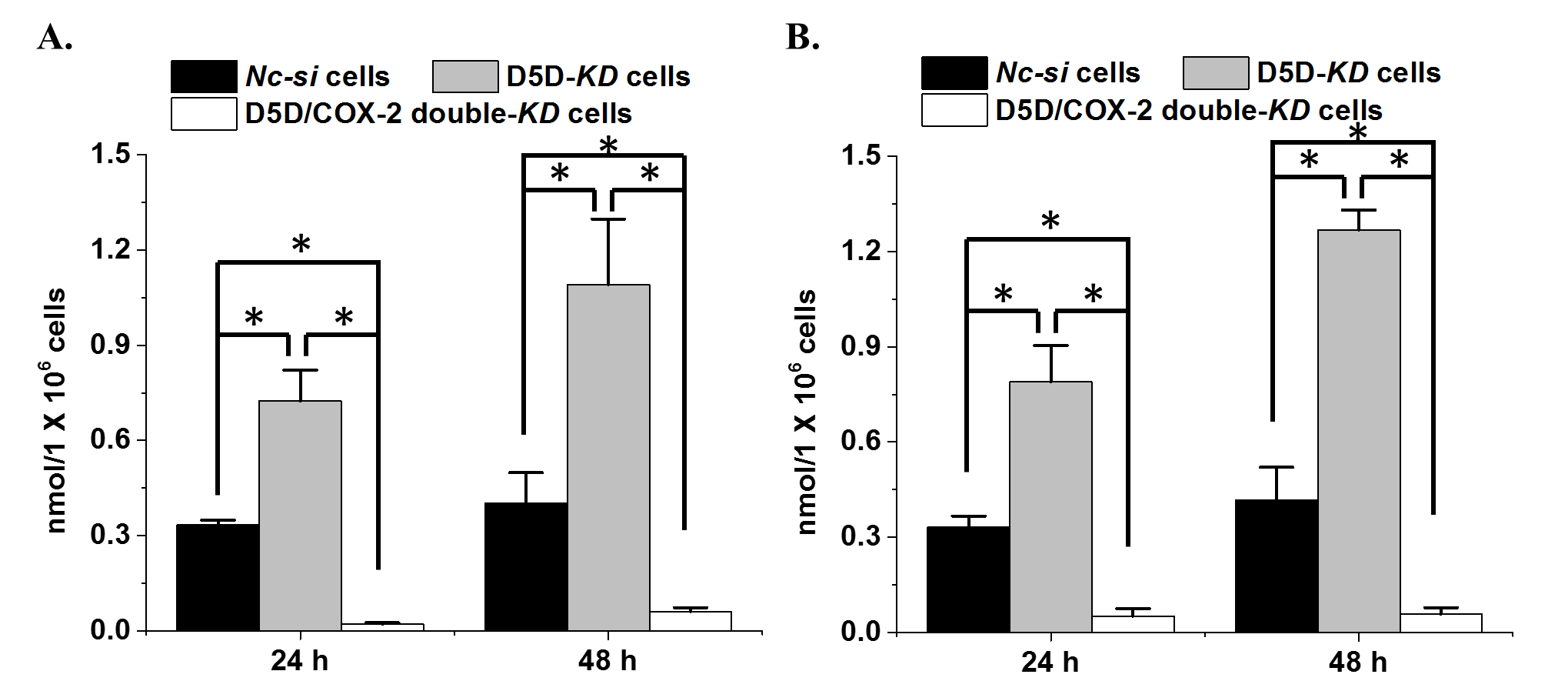
**
