# Supplementary material for: Knockdown delta-5-desaturase in breast cancer cells that overexpress COX-2 results in inhibition of growth, migration and invasion via a dihomo-γ-linolenic acid peroxidation dependent mechanism
Source: BMC Cancer. 2018 Mar 27;18:330. doi: 10.1186/s12885-018-4250-8 (PMC5870477; doi:10.1186/s12885-018-4250-8)
Supplement: Supplementary file 2 — Figure S2. Wound healing assays of NC-sh MDA-MB-231 and NC-sh 4 T1 cells upon DGLA treatment vs. controls. (DOCX 827 kb) [file 12885_2018_4250_MOESM2_ESM.docx]

**additional file 2**

**Method**

**Wound healing assay**

Cell migration of *Nc-sh* MDA-MB-231 and *Nc-sh* 4T1 cells upon DGLA treatment was assessed using same procedures as described in the manuscript.

**Figure Legend**

**Supplement Figure 2.** **A.** Wound healing assays of NC-sh MDA-MB-231 cells upon DGLA (100 µM, 48 h) treatment vs. controls (without DGLA); **B.** Wound healing assays of NC-sh 4T1 cells upon DGLA (100 µM, 48 h) treatment vs. controls (without DGLA, there is no significant statistical difference between controls and cells with DGLA treatment). Data represent as mean ± standard deviation.

**
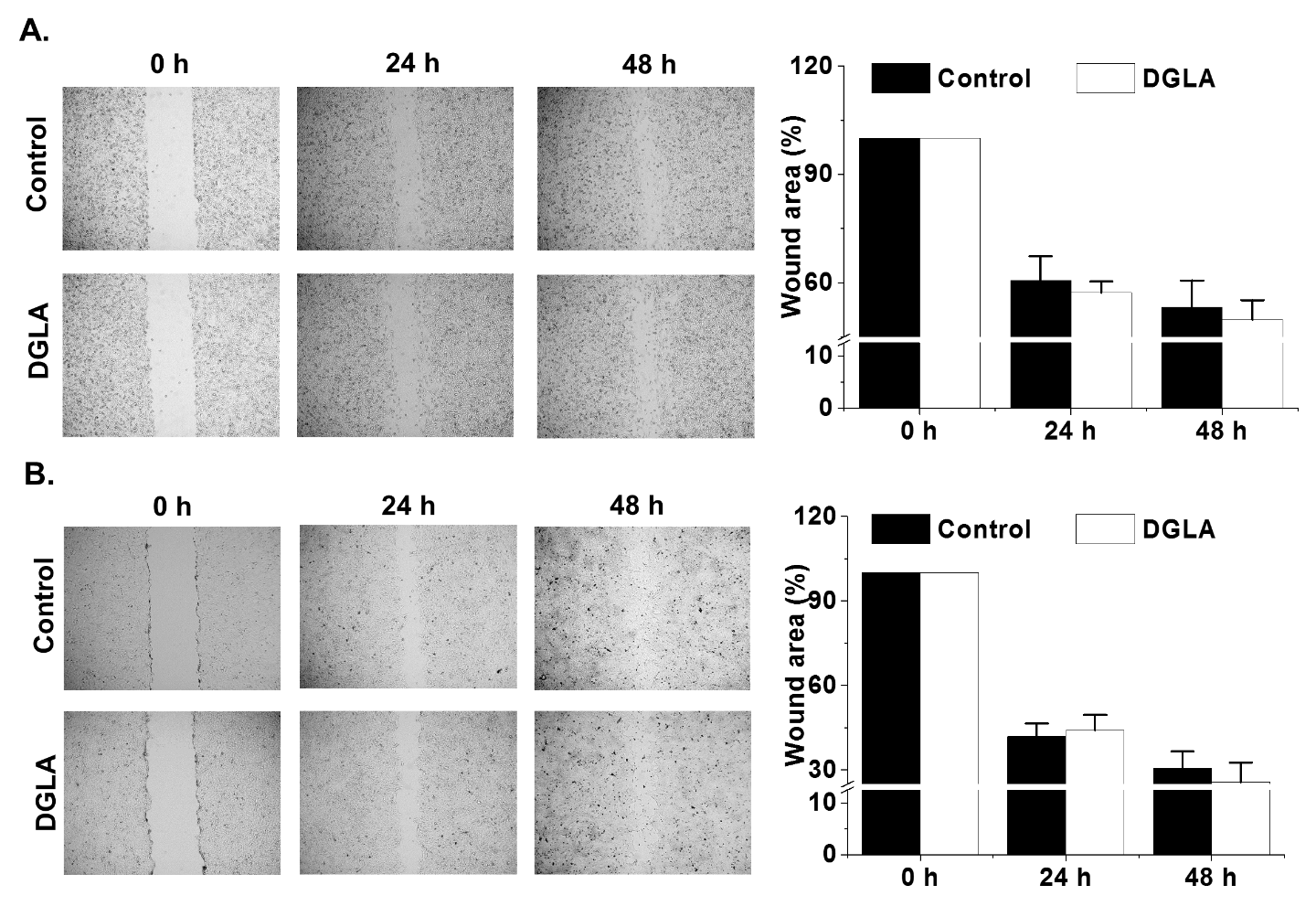
**
