# Supplementary material for: Knockdown delta-5-desaturase in breast cancer cells that overexpress COX-2 results in inhibition of growth, migration and invasion via a dihomo-γ-linolenic acid peroxidation dependent mechanism
Source: BMC Cancer. 2018 Mar 27;18:330. doi: 10.1186/s12885-018-4250-8 (PMC5870477; doi:10.1186/s12885-018-4250-8)
Supplement: Supplementary file 3 — Table S1. LC/MS quantification of DGLA and AA from Nc-si MB 231, D5D-KD MB 231, Nc-si 4 T1 or D5D-KD 4 T1 cells after DGLA treatment. (DOCX 17 kb) [file 12885_2018_4250_MOESM3_ESM.docx]

**additional file 3**

**Method**

**HDAC Activity Assay**

HDAC activity assay was measured using HDAC activity assay kit according to manufacturer’s instructions. Briefly, after cells were transfected with D5D- siRNA and treated with DGLA, nuclear proteins were extracted with NE-PER™ nuclear and cytoplasmic extraction reagents. Nuclear extracts were incubated with HDAC substrate at 37°C for 1 hr and then lysine developer was added to the mixture and incubated for 30 min at 37°C. The plate was read at 405 nm on a microplate reader. The HDAC activity in control cells without DGLA treatment was set to 100%.

**Figure Legend**

**Supplement Figure 3.** HDAC activity assay of D5D-KD 4T1 and D5D-KD MDA-MB-231 cells treated with vehicle or DGLA (100 µM). The HDAC activity in treatment group was calculated as a percentage to the control groups without DGLA treatment (normalized to 100%). Data represent as mean ± standard deviation. (*: significant difference with p < 0.05 from n ≥ 3).

**
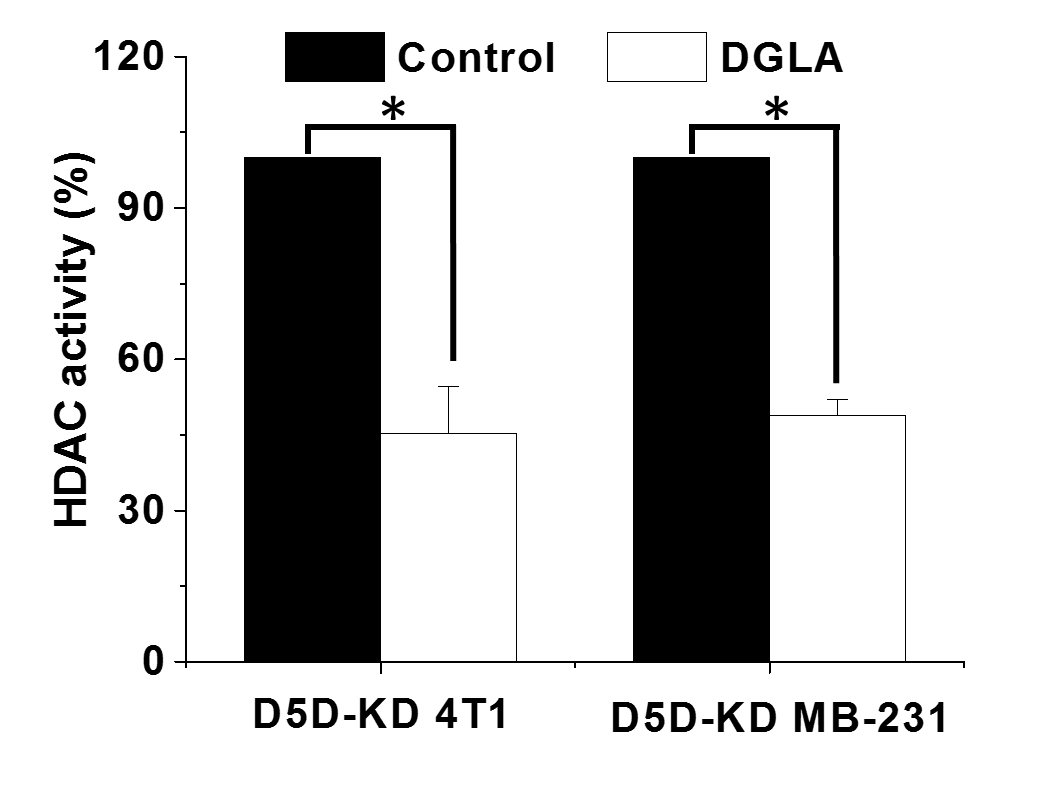
**
