# Supplementary material for: Knockdown delta-5-desaturase in breast cancer cells that overexpress COX-2 results in inhibition of growth, migration and invasion via a dihomo-γ-linolenic acid peroxidation dependent mechanism
Source: BMC Cancer. 2018 Mar 27;18:330. doi: 10.1186/s12885-018-4250-8 (PMC5870477; doi:10.1186/s12885-018-4250-8)
Supplement: Supplementary file 4 — Figure S3. HDAC activity assay of D5D-KD 4 T1 and D5D-KD MDA-MB-231 cells treated with vehicle or DGLA. (DOCX 36 kb) [file 12885_2018_4250_MOESM4_ESM.docx]

**additional file 4**

**Method**

**Detection of free DGLA and AA in cellular system**

The free DGLA and AA in D5D-*KD* MB 231, *NC-si* MB 231, D5D-*KD* 4T1 and *NC-si* 4T1 cells treated with 100 µM DGLA were quantified via LC/MS analysis. Briefly, 3.0×10^5^ cells per well in a 6-well plate were seeded overnight and transfected with D5D siRNA or negative control siRNA for 48 h. Then the cells were treated with 100 µM of DGLA (supplemented as 1.0 µL of ethanol solution into 1.0 mL of complete cell culture medium). At different time points, the cells (scratched off from well) with 1.0 mL of culture medium were collected, then mixed with 0.45 mL of methanol, 1.55 mL of water along with internal standards (DGLA-d_6_ and AA-d_8_). The mixture was vortexed for 1 min and set on ice for 30 min. After centrifuged for 15 min at 3,000 rpm, the supernatant of mixture was collected and adjusted to pH 3.0, then subjected to solid phase extraction (SPE) using a reverse phase SPE cartridge (SampliQ Silica C18 ODS, Agilent, CA, USA). Free fatty acids were eluted with 2.0 mL ethyl acetate from cartridge, the elution was vacuumed to dryness and reconstituted with 100 µL ethanol for LC/MS analysis.

An Agilent 1200 series HPLC system and Agilent 6300 LC/MSD SL ion trap mass was used to quantify the free fatty acids in reconstituted sample solution. LC separations were performed on a C18 column (Zorbax Eclipse-XDB, 4.6×75 mm, 3.5 μm) with 5.0 μL sample injection at a flow rate of 0.8 mL/min of gradient mobile phases (A: H_2_O-0.1% HOAc and B: ACN-0.1% HOAc): 0-12 min (isocratic), 68% A and 32% B; 12-14 min, 68 to 44% A and 32 to 56% B; 14-28 min (isocratic), 44% A and 56% B; 28-30 min, 44 to 14% A and 56 to 86% B; 30-38 min, 14 to 4% A and 86 to 95% B; and 38-44 min (isocratic), 5% A and 95% B. MS settings are as follows: electrospray ionization in negative mode; total ion current chromatograms in full mass scan mode (m/z 50 to m/z 600) were performed; nebulizer press, 15 psi; dry gas flow rate, 5.0 L/min; dry temperature, 325˚C; compound stability, 20%; number of scans, 50. The concentrations of DGLA were quantified using an internal standard curve by comparing the ratios of the peak areas of the DGLA to the peak areas of internal standard.

**table Legend**

**Supplement Table 1.** LC/MS quantification of DGLA and AA from cell medium containing 1.0×10^6^ of *Nc-si* MB 231, D5D-*KD* MB 231, *Nc-si* 4T1 or D5D-*KD* 4T1 cells after DGLA treatment (100 μM), respectively. Results were shown as mean ± SD with n ≥ 3.

| Cell Lines | DGLA (nmol in 1 × 10^6^ cells) | | | | AA (nmol in 1 × 10^6^ cells) | | | |
| --- | --- | --- | --- | --- | --- | --- | --- | --- |
|  | 8 h | 12h | 24 h | 48h | 8 h | 12h | 24 h | 48h |
| *Nc-si* MB231 | 30.27  ±1.65 | 12.06  ±6.67 | 6.49  ±1.61 | 1.03  ±0.62 | 0.43  ±0.06 | 0.36  ±0.04 | 0.36  ±0.08 | 0.38  ±0.05 |
| D5D-*KD* MB231 | 41.69  ±2.33 | 18.05  ±2.90 | 15.97  ±0.43 | 3.84  ±0.17 | 0.31  ±0.03 | 0.26  ±0.04 | 0.23  ±0.02 | 0.26  ±0.01 |
| *Nc-si*  4T1 | 36.86  ±0.68 | 16.99  ±0.44 | 7.37  ±0.13 | 1.90  ±0.03 | 0.44  ±0.03 | 0.29  ±0.02 | 0.33  ±0.03 | 0.35  ±0.02 |
| D5D-*KD* 4T1 | 45.73  ±6.98 | 21.37  ±0.43 | 14.03  ±0.17 | 4.81  ±0.15 | 0.32  ±0.05 | 0.15  ±0.01 | 0.20  ±0.03 | 0.22  ±0.03 |
